# Hot and Cold Spot Areas of Household Tuberculosis Transmission in Southern China: Effects of Socio-Economic Status and *Mycobacterium tuberculosis* Genotypes

**DOI:** 10.3390/ijerph16101863

**Published:** 2019-05-27

**Authors:** Zhezhe Cui, Dingwen Lin, Virasakdi Chongsuvivatwong, Edward A. Graviss, Angkana Chaiprasert, Prasit Palittapongarnpim, Mei Lin, Jing Ou, Jinming Zhao

**Affiliations:** 1 Department of Tuberculosis Control, Guangxi Zhuang Autonomous Region Center for Disease Control and Prevention, Nanning 530028, China; czz6997@163.com (Z.C.); drldw@163.com (D.L.); gxlinmei@126.com (M.L.); gxjfk@163.com (J.O.); yd0414zhjm@163.com (J.Z.); 2 Epidemiology Unit, Faculty of Medicine, Prince of Songkla University, Songkhla 90110, Thailand; 3 Department of Pathology and Genomic Medicine, The Center for Molecular and Translational Human Infectious Diseases Research, Houston Methodist Research Institute, Houston, TX 77030, USA; eagraviss@houstonmethodist.org; 4 Office for Research and Development, Faculty of Medicine Siriraj Hospital, Mahidol University, Bangkok 10700, Thailand; angkana.cha@mahidol.ac.th; 5 Department of Microbiology, Faculty of Science, Mahidol University, Bangkok 10700, Thailand; prasit.pal@mahidol.ac.th

**Keywords:** tuberculosis, genotypes, socioeconomic, transmission, household, contact

## Abstract

The aims of the study were: (1) compare sociodemographic characteristics among active tuberculosis (TB) cases and their household contacts in cold and hot spot transmission areas, and (2) quantify the influence of locality, genotype and potential determinants on the rates of latent tuberculosis infection (LTBI) among household contacts of index TB cases. Parallel case-contact studies were conducted in two geographic areas classified as “cold” and “hot” spots based on TB notification and spatial clustering between January and June 2018 in Guangxi, China, using data from field contact investigations, whole genome sequencing, tuberculin skin tests (TSTs), and chest radiographs. Beijing family strains accounted for 64.6% of *Mycobacterium tuberculosis* (Mtb) strains transmitted in hot spots, and 50.7% in cold spots (*p*-value = 0.02). The positive TST rate in hot spot areas was significantly higher than that observed in cold spot areas (*p*-value < 0.01). Living in hot spots (adjusted odds ratio (aOR) = 1.75, 95%, confidence interval (CI): 1.22, 2.50), Beijing family genotype (aOR = 1.83, 95% CI: 1.19, 2.81), living in the same room with an index case (aOR = 2.29, 95% CI: 1.5, 3.49), travelling time from home to a medical facility (aOR = 4.78, 95% CI: 2.96, 7.72), history of Bacillus Calmette-Guérin vaccination (aOR = 2.02, 95% CI: 1.13 3.62), and delay in diagnosis (aOR = 2.56, 95% CI: 1.13, 5.80) were significantly associated with positive TST results among household contacts of TB cases. The findings of this study confirmed the strong transmissibility of the Beijing genotype family strains and this genotype’s important role in household transmission. We found that an extended traveling time from home to the medical facility was an important socioeconomic factor for Mtb transmission in the family. It is still necessary to improve the medical facility infrastructure and management, especially in areas with a high TB prevalence.

## 1. Introduction

Tuberculosis (TB) continues to be a global concern because of its high infectivity, pathogenicity, mortality, and cost of therapy. In addition to the extra social and clinical burdens associated with TB disease, the patients’ family (and caregivers) are also at an increased risk of being infected with the *Mycobacterium tuberculosis* (Mtb) bacteria [1]. In 2017, the estimated number of TB cases in China was the second highest globally [2]. The realization of the End TB Strategy proposed by the World Health Organization (WHO) is directly related to the performance of TB control in China.

**Figure 1:**
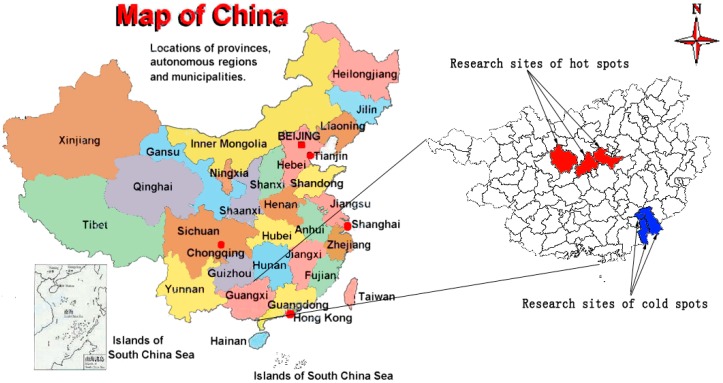
Research sites of hot and cold spots in Guangxi, China.

Guangxi Province is an autonomous region located in southern China. In addition to being one of the poorest provinces with the majority of the population being farmers, Guangxi is also regarded as a high TB notification region with 45,000 new TB cases reported annually by the National Notifiable Disease Reported System (NNDRS). Most TB patients come from rural areas where health resources are scarce [3]. From a previous spatiotemporal study, the TB notification rate in Guangxi was reported to be distributed unevenly at the county level [4]. Through the spatial autocorrelation analysis of our baseline survey in this study, we detected significantly high TB notification clusters (Moran’s I index between adjacent areas were close to 1) and significantly low zones (Moran’s I index between adjacent areas were close to −1) [5]. We defined the former as “hot spots” and the latter as “cold spots”. Based on this spatial feature, six counties in hot and cold spots were selected as the study sites as shown in Figure 1. In addition, the hot spot areas are inhabited by minorities, especially the Zhuang ethnic group, while the cold spot areas are inhabited mainly by the Han population (China’s main ethnic group). Some parts of China have reported a higher prevalence of TB in ethnic minority areas compared to areas where a majoring of the population are Han [6]. With this background, we have considered ethnicities as a potential risk factor in this study. Although there are large ethnic differences, by field investigation, we found that the living environment of residents in the two regions were similar and not considered overcrowded (about 30 m^2^ per family member).

Mtb strain-specific genomic diversity is an important factor in TB pathogenesis. Genomic diversity affects virulence, transmissibility, host response, and the emergence of drug-resistance. Of the seven major lineages of Mtb worldwide, the Beijing family strains belonged to Lineage 2 (the East-Asian lineage) and are widely prevalent in Eastern Asia, especially China [7,8]. Mycobacterial virulence plays an important role in the dissemination process of the TB disease [9]. Mtb Lineage 2, Lineage 3, and Lineage 4 strains are known to have virulent phenotypes compared to Mtb Lineage 1, Lineage 5, Lineage 6, and Lineage 7 [10]. Case-control studies and cohort studies have been conducted to analyze the characteristics of the Beijing family (Mtb Lineage 2.2) because of its association with drug resistance, high relapse rates, and pathogenicity [11,12,13]. However, there are few published studies about the Beijing family strain’s transmissibility in households. In addition, whole genome sequencing-based single-nucleotide polymorphisms (SNPs) as robust (stable) markers of genetic variation has been widely applied for phylogenetic analysis [14]. Due to the high resolution and stable inheritance, whole genome sequencing (WGS) is more discriminating than traditional Mtb genotyping methods such as mycobacterial interspersed repetitive unit-variable-number tandem repeat (MIRU-VNTR) typing and spatial oligonucleotide typing (spoligotyping) [10].

Mtb is transmitted from person to person via droplets. Therefore, people who are in close contact with TB patients are more likely to be infected with Mtb. A case-control study conducted in Ethiopia has shown that individuals that had a TB household member had an increased risk of developing TB by 3-fold [15]. Systematic TB contact tracing at the household level is not only an effective method to detect secondary cases of TB but the method is also an important epidemiologic tool to understand the transmission dynamics and prognostic factors of TB transmission in households [16,17]. Commonly used TB surveillance methods include a questionnaire survey, tuberculin skin test (TST) and chest imaging examination of household contacts of tuberculosis patients [17,18]. However, most studies ignore the influence of the bacteria with different genotypes and their interactions with the host’s environment on transmission.

With the increasing availability of WGS technology in China, it has become appropriate to determine which (and if) Mtb genotypes play a role in the intra-household transmission of TB disease in both hot and cold spots (areas with no clear pattern of spatial clustering). Thus, this study aimed to compare sociodemographic characteristics and Mtb genotype patterns among active TB cases in cold and hot transmission spots and quantify the influences of genotype, locality and potential determinants on the rates of LTBI among household contacts of index cases.

## 2. Methods

### 2.1. Ethics Approval

This study was approved by the Institutional Review Board of the Guangxi Center for Disease Control and Prevention (CDC) (GW-2017-0001) and the Research Ethics Committee of Prince of Songkla University (60-286-18-6). Both committees approved the informed consent procedure. All participants provided written informed consent to participate in this study.

### 2.2. Study Design

Two parallel case-contact studies in the cold and hot spot TB transmission areas of Guangxi Province were conducted from January to June 2018. We consecutively enrolled eligible pulmonary TB patients at county hospitals in these two areas and followed their household contacts. By comparing the different subgroups, in these study-targeted areas, we investigated the differences in the prevalence and transmissibility of Mtb at the household level.

### 2.3. Study Setting

Six Guangxi Province counties, including three “hot spots” and three “cold spots” of TB transmission, were identified based on TB notification spatial clustering analysis. Patients who were suspected as having pulmonary TB were confirmed at the county hospital with sputum smear, chest radiography and sputum culture. Only culture-positive TB cases were prospectively recruited. Cultured Mtb isolates were transported to the Guangxi CDC where Mtb DNA extraction occurred before shipping of mycobacterial DNA to Zeta Biosciences (Shanghai) for WGS. Clinical investigations of the TB patients and their contacts were conducted by a local hospital team under the supervision of Guangxi CDC. Clinical and laboratory data of the TB patients were routinely entered into the NNDRS which is overseen by Guangxi CDC. The local hospital team collected additional data from household contacts of TB cases by face-to-face interviews with household members after consent.

### 2.4. Study Participants and Selection Methods

#### 2.4.1. Sample Size

The sample size for household contacts was calculated based on the two independent proportions formula [19]. According to a previous study of major genotypes (Beijing strains) in Guangxi, the estimated proportion of the Beijing family genotype was 70% in hot spot areas and 50% in cold spot areas [20]. With a type I error of 0.05 and a power of 90%, at least 124 active TB cases in each area were required. We estimated that 310 household contacts in each area would be recruited based on the screening rate from a pilot study.

#### 2.4.2. Eligibility Criteria for Index Cases

All individuals confirmed as having pulmonary TB at the county hospital of hot and cold spot areas were recruited, and eligible index TB cases must have been a resident in one of six counties for at least two years prior to TB case identification. Individuals with TB who were unable to communicate with research staff were excluded.

#### 2.4.3. Whole Genome Sequencing and Genotyping Performance

At the Guangxi CDC, a genetic sample kit (HiPure Bacterial DNA Kit, Magen Biotech Co. Ltd., Xuzhou, China) was used for Mtb DNA extraction. At Zeta Biosciences (Shanghai, China), next generation sequencing library preparations were constructed following the manufacturer’s protocol (Illumina TruSeq DNA Nano Library Prep Kit, Illumina, Inc., San Diego, CA, USA). For each sample, 100 ng DNA was randomly fragmented to <500 bp by sonication (Covaris S220, Covaris, Inc., Woburn, MA, USA). The fragments were treated with End Prep Enzyme Mix for end repairing and with A-Tailing Mix for dA-tailing, followed by a T-A ligation to add adaptors to both ends. Size selection of Adaptor-ligated DNA was then performed using SPB Beads, and fragments of ~410 bp (with the approximate insert size of 350 bp) were recovered. Each sample was then amplified by PCR for eight cycles using P5 and P7 primers, with both primers carrying sequences which can anneal with flowcell to perform a bridge PCR and P7 primer carrying a six-base index allowing for multiplexing. The PCR products were cleaned up using SPB Beads, validated using an Agilent 2100 Bioanalyzer (Agilent Technologies, Palo Alto, CA, USA) and quantified by Qubit2.0 Fluorometer (Invitrogen, Carlsbad, CA, USA). Libraries with different indices were subsequently multiplexed and loaded on an Illumina HiSeq instrument according to the manufacturer’s instructions (Illumina, San Diego, CA, USA). Sequencing was carried out using a 2 × 150 paired-end (PE) configuration; image analysis and base calling were conducted by the HiSeq Control Software (HCS) + OLB + GAPipeline-1.6 (Illumina) on the HiSeq instrument. *Mycobacterium tuberculosis* WGS data files in the FASTQ format of each sample were trimmed with Trimmomatic v0.3.2. Trimmomatic cut bases off at the start of a read if the quality score was under 3 and retained reads at a minimal length of 91 bp. Trimmed paired-end reads ware aligned using Bowtie2 v2.3.0 against the genome of Mycobacterium tuberculosis H37Rv (Genbank accession: AL123456.3) with the parameter “--very-sensitive”. SNPs were identified using VarScan v2.3.8 and SAMtools v1.3.1; variations with a coverage under 30× or the minimal frequency of the homozygote below 0.99 were ignored. Further filter-ware was applied to all the VCF files using bcftools v1.9. In the filter criteria, heterozygous SNP or SNP within 5 bp of an indel was filtered. SNPs with a genotype quality below 255 or mapped alleles depth under 100× were also discarded. Variations in the coding sequence of proteins that contain proline-glutamate or proline-proline-glutamate motifs were characterized by Genebank’s annotation and filtered from downstream analysis. Finally, 26,869 SNPs remained after filtering. VCF-kit was use to concentrate all the remaining SNPs into pseudo-sequences for phylogeny analysis. A maximum-likelihood phylogeny was reconstructed using RAxML v8.1.9 with a general time-reversible (GTR) nucleotide substitution model and 100 bootstrap replicates; the GAMMA model of rate heteorgeneity was estimated by RAxML itself [21]. Finally, the crude and filtered fasta-files, vcf files, spleen necrosis virus matrix, classified spoligotype and lineage genotype data, and the gene structure difference coefficient (Fst) between hot and cold spot areas of TB were delivered to the Guangxi CDC for downstream data analysis.

#### 2.4.4. Household Contacts

Following consent of the index TB cases and their family members, contact tracing was conducted on immediate household contacts of index TB cases if they lived with the index TB case for more than three months. Children under the age of five were excluded. For interviewees with limited understanding, their immediate family members were used as a proxy. In order to screen for active TB among household contacts, a Mantoux TST and chest radiography were used in parallel.

#### 2.4.5. Tuberculin Skin Tests for Mtb Infection

As recommended by China’s National Tuberculosis Control Program, the TST was carried out by the Mantoux method injecting a 5 tuberculin unit dose of BCG purified protein derivative (Beijing Sanroad Biological Products Co. Ltd., Beijing, China) into the volar surface of the forearm. The TST injection was performed by trained nurses from the local TB clinic and was made with a 1-mL tuberculin syringe with the needle bevel facing upward. Identification marks for the furthest induration points from the inoculation site were made on the skin. The TST reaction was read between 48 and 72 h by TB clinic doctors. A patient who did not return within 72 h was given a reappointment for another TST. A patient with an average diameter of induration ≥10 mm was considered as an Mtb-infected individual [22,23,24].

#### 2.4.6. Secondary and LTBI Identification

TST and chest radiography positive subjects with suspected TB symptoms were identified and recorded and their sputa were sent for laboratory examination. To confirm TB diagnosis, the study team used a sputum alpha-fetoprotein smear and culture with drug sensitivity testing and a genotype test. Characteristic features of the index cases were analyzed in relation to active TB positivity results. Household contacts who had TSTs with an induration ≥10 mm and who were diagnostically excluded from the possibility of active TB were identified as having latent TB infection.

### 2.5. Variables and Data Collection

Household-level variables obtained from TB cases and their household contacts included: the average age and body mass index (BMI) of all contacts, number of family members, size of living area, the average diameter of induration, and the TST positive rate. Individual variables included: occupation, ethnicity, education status, marital status, duration of living at the present address, income, history of household contact with the TB patient, history of smoking, history of drinking, history of BCG vaccination, travel history, travelling time from home to medical facility, TB symptoms, severity of TB symptoms, delay in TB diagnosis, BMI, other chronic or immunocompromising diseases, HIV status, and diabetes mellitus status (self-report). Structured and piloted questionnaires were used to collect all information about individuals and household-level data.

### 2.6. Data Management and Analysis

Epi Data (version 3.1) was used for double entering of the data at regular intervals while R (v 3.3.2) was used for data management and analysis. MEGA-X (v 10.0.5) and Fig Tree (v 1.4.4) were used for editing phylogenetic trees.

Univariate analysis was initially used to assess the differences of variables between hot and cold spot areas and Mtb-infected individuals versus No-Mtb infected individuals. Variables with *p*-values < 0.20 [25] and those deemed as important covariates were included in a multivariate mixed-effects model in which the household identifier was the clustering unit. Statistical significance was determined by using a *p*-value < 0.05.

## 3. Results

**Figure 2:**
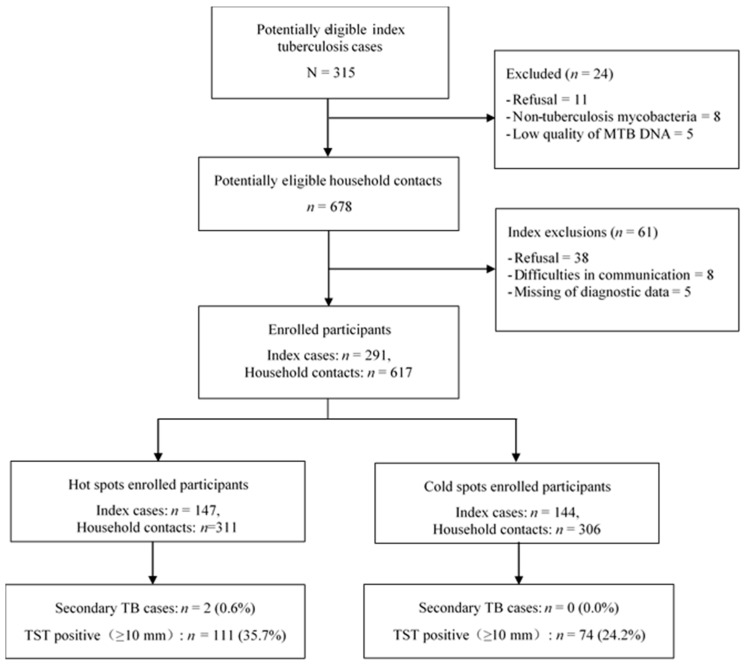
Profile of household Mtb transmission study.

A total of 315 potentially eligible index TB cases with complete diagnostic data were identified. Twenty-four TB cases were excluded due to refusal to participate, non-tuberculosis mycobacteria (NTM) or low quality of Mtb DNA (Figure 2). Of the remaining 291 enrolled TB index patients and their 761 household contacts, we excluded 144 because of refusal to participate, difficulties in communication and lack of diagnostic data. Consequently, we included 291 index cases (147 from hot spot areas and 144 from cold spot areas) and their 617 household contacts (311 from hot spot areas and 306 from cold spot areas). Two secondary TB cases and 185 TST positive household contacts were detected in this study. The maximum induration size in TST positive cases was 23 mm.

### 3.1. Characteristics of Index Cases and Household Contacts

Table 1 shows a comparison of various county and individual level factors between the two geographical areas. We identified three hot spot counties having a population of 1.5 million and three cold spot counties with a population of 2.7 million. Counties in hot spot areas had a higher prevalence of multi-drug resistant TB and a higher mortality rate than those in cold spot areas, but had a lower per capita gross domestic product, percentage of Han ethnicity, and number of hospitals. At the individual level, hot spot areas had a higher proportion of elderly, Zhuang minority, less educated, low-income earners, and underweight TB cases who had a delay in their diagnosis and sputum smear with more than 2+ strong smear-positive cases.

Table 2 summarizes the characteristics of household contacts of index TB cases. Ethnicity, education level, and income of family contacts were consistent with the index case. In addition, migrant work, living with the index cases, having suspected TB symptoms and a positive TST (induration ≥ 10 mm), and having a higher percentage of TB cases in hot spot areas were significantly high (*p* < 0.05). Table 3 compares the aggregated values of characteristics of the households of the two areas. The average diameter of induration and the positive TST rate in the household were significantly higher in the hot spots (*p* < 0.05).

### 3.2. Comparison of the Mtb Genotypes Between Hot and Cold Spots of TB

**Figure 3:**
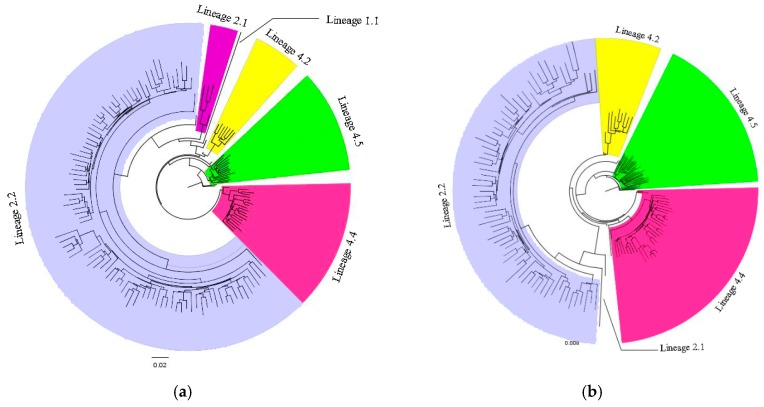
Phylogenetic tree of Mtb strains found in hot and cold spot counties. (**a**) Hot spot counties; (**b**) Cold spot counties.

A phylogenetic tree of genotypic information from the Mtb isolates for hot and cold spot counties is shown in Figure 3. The major Mtb lineages were Lineage 2 and Lineage 4. Lineage 2.2 represents the Beijing family strain which accounted for 64.6% in hot spots and 50.7% in cold spots (*p* = 0.02). The overall proportion of Mtb Lineage 4 was higher in cold spot counties.

### 3.3. Independent Effects of Locality and Genotypes of the Index Cases on TST Result

Table 4 summarizes the univariate analysis of factors associated with TST status. Living in a hot spot area, being the spouse of an index case, living in the same room as an index case, having a BCG vaccination, Beijing family genotype, delay in TB diagnosis, and old age were significantly associated with being TST positive.

Table 5 compares models with different independent variable groups included in an incremental fashion. Model 1 contains only the spatial effect. Model 2 adds the Mtb genotype (Beijing vs. non-Beijing families) of the index cases to adjust for the spatial effect. Model 3 added socio-economic factors to test for confounding. Model 4 introduced potential influencing factors such as living in the same room as an index case, BCG vaccination status, and delay in diagnosis of index TB case. Based on the Akaike Information Criterion (AIC) judgment [26], Model 4 was determined as the best fitting model. In all models, both the spatial location (aOR = 1.61) and the genotype of the index case (aOR = 1.83) retained statistical significance throughout. Adding variables such as travelling time from home to a medical facility ≥ 1 h (aOR = 4.78), living in the same room with an index case (aOR = 2.29), BCG vaccination status (aOR = 2.02), and delay in diagnosis of the index case (aOR = 2.56) were independently associated with TST status.

## 4. Discussion

TB cases from hot spot county areas were more likely to belong to a minority ethnic group, have a lower socioeconomic status, be in poor physical condition, and have a delay in diagnosis compared to those from cold spot areas. The proportion of cases having the Beijing family genotype (64.6%) in hot spot areas was significantly higher than that in cold spot areas (50.7%). The TST positive rate among household contacts in the hot spot areas was 35.7%, significantly higher than that in cold spot areas (24.2%); and with multivariate analysis, it remained significantly associated with the location. Genotype (Beijing vs. non-Beijing) was also significantly associated with TST status even after adjusting for travelling time from home to medical facility, BCG vaccination status and delay in diagnosis. Income and education had no effect on TST results. Ethnicity, however, lost its significance due to the confounding effect of the travelling time.

The association between TB and socio-economic status has been well documented [27,28,29,30,31,32]. The hot spot areas were predominated by those individuals with low socioeconomic status, which we first believed to be the explanation for Mtb transmission. We did not find support for the association because income, education and occupation were not associated with the household transmission, and ethnicity lost its significant effect after adjustment for travelling time and other potential influencing factors. Thus, travelling time might be the final common path of causation between socio-economic status and TB household transmission (aOR = 4.78). The long travelling time from home to medical facilities implicates poor access to health services. In particular, family members with active tuberculosis may become super-spreaders, spreading mycobacterium tuberculosis in their household, due to their failure to receive timely diagnosis and standard treatment. Thus, this factor increases the local TB burden. Our finding at the county level supported this hypothesis. Cold spot county areas had three times as many medical facilities as the hot spot county areas. This predictor is consistent with a household tracing study conducted in Tunisia [33].

In addition to a long travelling time, other host factors were also associated with Mtb intra-household transmission in this study. Delay in TB diagnosis of the index TB case provided a long-term exposure of Mtb to the household contacts (aOR = 2.56). Studies conducted in Sudan and western China had similar results [34,35]. Most of the active TB patients in the hot spot areas still shared a bedroom with household contacts. This behavior increased the chance of TB infection (aOR = 2.29) due to the limited living areas of households and low awareness of TB prevention by the inhabitants. This predictive factor has also been reported by Jones-Lopez in a study from Brazil [36].

The ability of specific Mtb pathogens to disseminate plays a critical role in the TB epidemic [37]. Our analysis confirmed that the Beijing family genotype strains in the index TB cases were more prevalent in areas where spatial clustering was high. Beyond that, this strain increased the risk of household TB transmission compared to non-Beijing genotypes after adjusting for other risk factors (aOR = 1.83). The Beijing genotype family is widespread in Asian countries and also in the United States [38,39]. These strains are considered to be related to immune escape from BCG protection, increasing virulence, drug resistance, and transmissibility [40]. Beijing genotype strains have been shown to be a TB complex associated with drug-resistance, which is more common in hot spot areas [41]. Compared with northern China, the distribution of Beijing strains in our two research sites is relatively low [42,43,44]. The level of importance of the TB complex on transmission, therefore, varies across different cofactors such as the environment and population mobility. Guangxi is adjacent to southeast Asian countries, and frequent economic and cultural communication with these countries may also lead to the exchanges of different Mtb lineages, and thus, the genomic polymorphism becomes greater.

The detection rate of LTBI in the hot spot county areas was 35.7%, higher than that in cold spot county areas (24.2%) and other regions with low TB incidence [45,46]. After controlling for other factors, the geographic location proved to be an independent factor for household Mtb transmission (aOR = 1.61). This significant effect could not be explained by other variables that were included in the model. In the hot spot county areas, these TB transmission promotors from outside of the household may be more common, but this supposition is unclear in the current study. However, more unknown mechanisms outside the household would need further studies.

A TST (induration ≥ 10 mm) was used to investigate LTBI among household contacts in the current study. The TST result may be difficult to distinguish between an Mtb infection, vaccination of BCG, and non-tuberculosis mycobacteria infection, and has low specificity. However, from a meta-analysis of studies in mainland China, the non-tuberculosis mycobacteria infection rate was about 6.3% (range: 5.4–7.4%) in tuberculosis suspected population. In our study, the non-tuberculosis mycobacteria detection rate was only 2.7% (8/291). Thus, it is unlikely that the exposure of non-tuberculosis mycobacteria affected TST specificity. Studies from China, Germany, and Australia identified that the main reason affecting TST judgment of LTBI was BCG vaccination [47,48,49]. The results of this study also support this viewpoint (aOR = 2.02) and are similar to studies from Singapore and Gambia [50,51]. Interferon-gamma release assay (IGRAs) is an alternative method for LTBI diagnosis. However, due to the high cost of testing, it is still not widely available in high-epidemic, developing countries. Thus, WHO has declared that in areas of high TB prevalence, IGRAs should not replace the TST for identification of individuals with LTBI [22,52].

In this study, we could not obtain Mtb isolates from household contacts and have not determined the transmission dynamics outside the household. The relationships involving Mtb transmission outside the household are known to be associated with environmental factors and should be taken into account in future studies.

## 5. Conclusions

The findings of this study confirmed the strong transmissibility of the Beijing genotype family strains and this genotype’s important role in household transmission. We found that an extended traveling time from home to the medical facility was an important socioeconomic factor for Mtb transmission in the family which is consistent with a study conducted in the Yunnan Province of China in 2005 [35]. Over the past decade, in most districts of Guangxi, the diagnosis and treatment of TB has shifted from county-level CDC clinics to county-level designated hospitals having advanced diagnostic equipment, but poor access to medical facilities remains a significant risk factor for Mtb household transmission. Thus, it is still necessary to improve the medical facility infrastructure and management, especially in areas with a high TB prevalence.

## Figures and Tables

**Table 1:** Comparison of characteristics of TB index cases between cold and hot spot areas.

Variable	Cold Spots (*n*, %)	Hot Spots (*n*, %)	*p-*Value
**County-level**			
Number of counties	3	3	
GDP per capita (Renminbi)	35,268	17,556	
Proportion of Han ethnicity (%)	97.67	31.67	
Prevalence of MDR-TB (%)	0.20	1.69	
Mortality rate	0.08	0.14	
Number of hospitals	29	10	
**Individual-level**			
Total number	144 (100.0)	147 (100.0)	
Gender			0.740
Male	114 (79.2)	113 (76.9)	
Female	30 (20.8)	34 (23.1)	
Age group (years)			0.009
<20	7 (4.9)	5 (3.4)	
20–29	21 (14.6)	5 (3.4)	
30–39	26 (18.1)	19 (12.9)	
40–49	21 (14.6)	27 (18.4)	
50–59	24 (16.7)	35 (23.8)	
≥60	45 (31.2)	56 (38.1)	
Ethnicity			<0.001
Han	139 (96.5)	5 (3.4)	
Zhuang	4 (2.8)	130 (88.4)	
Others	1 (0.7)	12 (8.2)	
Education			<0.001
None	2 (1.4)	18 (12.2)	
Primary school	45 (31.2)	66 (44.9)	
Middle school	52 (36.1)	44 (29.9)	
High school	38 (26.4)	19 (12.9)	
University	7 (4.9)	0 (0)	
Occupation			0.056
Farmer	101 (70.1)	116 (78.9)	
Migrant worker	14 (9.7)	14 (9.5)	
Student	10 (6.9)	1 (0.7)	
Civil servant	1 (0.7)	2 (1.4)	
Others	18 (12.5)	14 (9.5)	
Marital status			0.435
Married	112 (77.8)	123 (83.7)	
Single	26 (18.1)	19 (12.9)	
Others	6 (4.2)	5 (3.4)	
Income per month (Renminbi)			<0.001
<3000	100 (69.4)	133 (90.5)	
3000–4000	21 (14.6)	12 (8.2)	
4000–5000	17 (11.8)	1 (0.7)	
>5000	6 (4.2)	1 (0.7)	
Number of TB patients in family			0.082
None	132 (91.7)	124 (84.4)	
One or more	12 (8.3)	23 (15.6)	
History of smoking			0.696
Never	73 (50.7)	70 (47.6)	
Former	48 (33.3)	48 (32.7)	
Current	23 (16)	29 (19.7)	
History of drinking alcohol			0.289
Never	98 (68.1)	87 (59.2)	
Former	28 (19.4)	36 (24.5)	
Current	18 (12.5)	24 (16.3)	
HIV status			0.918
Negative	136 (94.4)	139 (94.6)	
Positive	2 (1.4)	1 (0.7)	
Unknown	6 (4.2)	7 (4.8)	
Diabetes status			0.134
Negative	122 (84.7)	111 (75.5)	
Positive	21 (14.6)	33 (22.4)	
Unknown	1 (0.7)	3 (2)	
BCG vaccination history			0.964
Unvaccinated	32 (22.2)	34 (23.1)	
Vaccinated	112 (77.8)	113 (76.9)	
BMI (kg/m^2^)			<0.001
<18	11 (7.6)	39 (26.5)	
18–24.99	127 (88.2)	101 (68.7)	
≥25	6 (4.2)	7 (4.8)	
Outside travel greater than 3 months			0.017
No	110 (76.4)	129 (87.8)	
Yes	34 (23.6)	18 (12.2)	
Severity of TB symptoms			0.587
Median (IQR)	2 (1,2)	2 (1,2)	
Delay of TB Diagnosis			0.038
Yes	121 (82.6)	136 (92.5)	
No	23 (17.4)	11 (7.5)	
Retreat			0.135
No	127 (88.2)	138 (93.9)	
Yes	17 (11.8)	9 (6.1)	
Smear result			<0.001
Median (IQR)	2+ (1+,2+)	3+ (2+,3+)	
Travelling time from home to medical facility			0.103
Greater than one hour	53 (36.8)	40 (27.2)	
Within one hour	91 (63.2)	107 (72.8)	

TB: Tuberculosis. GDP: Gross domestic product. IQR: Inter-quartile range. MDR-TB: Multi-drug resistant tuberculosis.

**Table 2:** Comparison of characteristics of the household contacts between cold and hot spot transmission areas.

Variables	Cold Spots *n* = 306	Hot Spots *n* = 311	*p*-Value
Relationship with index cases			0.819
Non-spouse	220 (71.9)	220 (70.7)	
Spouse	86 (28.1)	91 (29.3)	
Live together with index cases			<0.001
Live separate	196 (64.1)	126 (40.5)	
Live in same room	110 (35.9)	185 (59.5)	
Local residence			0.725
Local	303 (99)	306 (98.4)	
Immigration	3 (1)	5 (1.6)	
Gender			0.605
Male	152 (49.7)	147 (47.3)	
Female	154 (50.3)	164 (52.7)	
Age group (years)			0.886
5–14	39 (12.7)	44 (14.1)	
15–40	103 (33.7)	104 (33.4)	
40–59	106 (34.6)	100 (32.2)	
≥60	58 (19)	63 (20.3)	
Ethnicity			<0.001
Han	298 (97.4)	31 (10)	
Zhuang	8 (2.6)	247 (79.4)	
Others	0 (0.0)	33 (10.6)	
Education			<0.001
None	9 (2.9)	37 (11.9)	
Primary school	114 (37.3)	128 (41.2)	
Middle school	113 (36.9)	100 (32.2)	
High school	51 (16.7)	31 (10)	
University	19 (6.2)	15 (4.8)	
Occupation			<0.001
Farmer	211 (69.0)	164 (52.7)	
Migrant worker	16 (5.2)	37 (11.9)	
Student	42 (13.7)	62 (19.9)	
Civil servant	5 (1.6)	6 (1.9)	
Others	32 (10.5)	42 (13.5)	
Marital status			0.179
Married	201 (65.7)	205 (65.9)	
Single	84 (27.5)	73 (23.5)	
Others	21 (6.9)	33 (10.6)	
Income per month (Renminbi)			0.011
≤3000	225 (73.5)	262 (84.2)	
3001–4000	55 (18)	33 (10.6)	
4001–5000	22 (7.2)	13 (4.2)	
>5000	4 (1.3)	3 (1)	
History of smoking			0.201
Never	250 (81.7)	236 (76.9)	
Former	18 (5.9)	17 (5.5)	
Current	38 (12.4)	54 (17.6)	
History of drinking			0.125
Never	259 (84.6)	239 (78.4)	
Former	10 (3.3)	12 (3.9)	
Current	37 (12.1)	54 (17.7)	
HIV status			0.778
Positive	1 (0.3)	1 (0.3)	
Negative	278 (90.8)	287 (92.3)	
Unknown	27 (8.8)	23 (7.4)	
Diabetes status			0.955
Negative	280 (91.5)	285 (91.6)	
Positive	25 (8.2)	24 (7.7)	
Unknown	1 (0.3)	2 (0.6)	
BCG vaccination history			0.499
Unvaccinated	46 (15)	54 (17.4)	
Vaccinated	260 (85)	257 (82.6)	
BMI (kg/m^2^)			0.708
<18	31 (10.1)	35 (11.3)	
18–24.99	260 (85)	257 (82.6)	
≥25	15 (4.9)	19 (6.1)	
Travelling time from home to medical facility			0.076
Greater than one hour	110 (35.9)	90 (28.9)	
Within one hour	196 (64.1)	221 (71.1)	
TST (induration, mm)			0.014
<5	202 (66)	179 (57.6)	
5–9.9	28 (9.2)	21 (6.8)	
10–14.9	66 (21.6)	88 (28.3)	
≥15	10 (3.3)	23 (7.4)	
Suspected TB symptoms			0.029
No	301 (98.4)	295 (94.9)	
Yes	5 (1.6)	16 (5.1)	
Diagnosis of TB			0.499
Non-TB	306 (100)	309 (99.4)	
TB	0 (0)	2 (0.6)	

HIV: Human immunodeficiency virus. BMI: Body mass index. TST: Tuberculin skin test. TB: Tuberculosis.

**Table 3:** Comparison of the household-level variables between cold and hot spot areas.

Variable	Cold Spots *n* = 144	Hot Spots *n* = 147	*p-*Value
Number of contacts per household (*n*)	2 (2, 3)	2 (1, 3)	0.810
TST screening rate (%)	100 (66.7, 100)	100 (66.7, 100)	0.585
Percentage of children per household (%) *	0 (0, 0)	0 (0, 0)	0.435
Average age (years)	41.5 (34.8, 52.8)	44.3 (34.4, 54.5)	0.271
Average BMI	20.9 (19.8, 21.9)	20.7 (19.7, 21.7)	0.597
Percentage of smokers (%)	0 (0, 30)	0 (0, 30)	0.152
Per capita size of living area (m^2^)	30 (20, 40)	30 (20.6, 42.7)	0.952
Average diameter of induration (mm)	2.7 (0, 6.2)	4.7 (0, 7.8)	0.005
Positive rate of TST (%)	0 (0, 50)	0.3 (0, 50)	0.023

Numbers in table are median (inter-quartile range). TST: Tuberculin skin test. IQR: Inter-quartile range. * Children older than five years of age.

**Table 4:** Univariate analysis of factors associated with TST status among TB household contacts.

Variables	TST (−) (*n*, %)	TST (+) (*n*, %)	*p*-Value
Total	432	185	
Locality			0.002
Cold spots	232 (53.7)	74 (40)	
Hot spots	200 (46.3)	111 (60)	
Suspected TB Symptoms			1.000
No	417 (96.5)	179 (96.8)	
Yes	15 (3.5)	6 (3.2)	
TB status			0.510
Non-TB	431 (99.8)	184 (99.5)	
TB	1 (0.2)	1 (0.5)	
Relationship with the index case			0.005
Non-spouse	323 (74.8)	117 (63.2)	
Spouse	109 (25.2)	68 (36.8)	
Lives in the same room with the index case			<0.001
No	250 (57.9)	72 (38.9)	
Yes	182 (42.1)	113 (61.1)	
Gender			0.465
Male	214 (49.5)	85 (45.9)	
Female	218 (50.5)	100 (54.1)	
Age group (years)			0.145
<40	197 (45.6)	93 (50.3)	
≥60	74 (17.1)	38 (20.5)	
40–59	161 (37.3)	54 (29.2)	
Ethnicity			0.452
Han	234 (54.2)	95 (51.4)	
Others	20 (4.6)	13 (7)	
Zhuang	178 (41.2)	77 (41.6)	
Education			0.592
None	36 (8.3)	10 (5.4)	
Primary school	167 (38.7)	75 (40.5)	
Middle school	151 (35)	62 (33.5)	
High school	57 (13.2)	25 (13.5)	
University	21 (4.9)	13 (7)	
Occupation			0.617
Civil servant	8 (1.9)	3 (1.6)	
Farmer	268 (62)	107 (57.8)	
Migrant worker	34 (7.9)	19 (10.3)	
Others	54 (12.5)	20 (10.8)	
Student	68 (15.7)	36 (19.5)	
Marital status			0.599
Married	288 (66.7)	118 (63.8)	
Single	105 (24.3)	52 (28.1)	
Others	39 (9)	15 (8.1)	
Income per month (Renminbi)			0.507
≤3000	336 (77.8)	151 (81.6)	
3001–4000	62 (14.4)	26 (14.1)	
4001–5000	28 (6.5)	7 (3.8)	
>5000	6 (1.4)	1 (0.5)	
TB patients in family			0.808
No	300 (69.4)	131 (70.8)	
Yes	132 (30.6)	54 (29.2)	
History of smoking			0.522
Never	336 (78.3)	150 (81.5)	
Former	24 (5.6)	11 (6)	
Current	69 (16.1)	23 (12.5)	
History of drinking alcohol			0.808
Never	349 (81.7)	149 (81)	
Former	14 (3.3)	8 (4.3)	
Current	64 (15)	27 (14.7)	
HIV status			0.188
Positive	1 (0.2)	1 (0.5)	
Negative	391 (90.5)	174 (94.1)	
Unknown	40 (9.3)	10 (5.4)	
Diabetes status			0.944
Negative	395 (91.4)	170 (91.9)	
Positive	35 (8.1)	14 (7.6)	
Unknown	2 (0.5)	1 (0.5)	
BCG vaccination history			0.024
Unvaccinated	80 (18.5)	20 (10.8)	
Vaccinated	352 (81.5)	165 (89.2)	
BMI (kg/m^2^)			0.591
<18	48 (11.1)	18 (9.7)	
18–24.99	358 (82.9)	159 (85.9)	
≥25	26 (6)	8 (4.3)	
Outside travel greater than 3 month			0.599
No	364 (85)	151 (83)	
Yes	64 (15)	31 (17)	
Travelling time from home to medical facility			<0.001
Greater than one hour	332 (76.9)	85 (45.9)	
Within one hour	100 (23.1)	100 (54.1)	
Mtb genotype of the index case			<0.001
Non-Beijing	195 (45.1)	53 (28.6)	
Beijing	237 (54.9)	132 (71.4)	
Gender of the index case			0.302
Male	325 (75.2)	147 (79.5)	
Female	107 (24.8)	38 (20.5)	
The age group of the index case			0.035
<40	136 (31.5)	41 (22.2)	
40–59	140 (32.4)	76 (41.1)	
≥60	156 (36.1)	68 (36.8)	
Delay for index TB diagnosis			0.002
Yes	369 (85.4)	175 (94.6)	
No	63 (14.6)	10 (5.4)	

TB: Tuberculosis. Mtb: *Mycobacterium tuberculosis*. HIV: Human immunodeficiency virus. BMI: Body mass index. TST: Tuberculin skin test.

**Table 5:** Results of multivariate analysis of factors associated with tuberculin skin test result (aOR 95% CI).

Variable	Model 1	Model 2	Model 3	Model 4
Spatial area	1.75 (1.22, 2.50)	1.57 (1.09, 2.25)	2.70 (1.12, 6.51)	1.61 (1.05, 2.45)
Index genotype		1.90 (1.29, 2.79)	1.93 (1.25, 2.97)	1.83 (1.19, 2.81)
SES (Ethnicity, Income, Education, Occupation, Marital status, Travelling time from home to the medical facility)			Long travelling: 4.71 (2.84, 7.81)	Long travelling: 4.78 (2.96, 7.72)
Others (Lives in the same room as the index case, history of BCG vaccination, delay in diagnosis)				Same room: 2.29 (1.50, 3.49) BCG vaccinated: 2.02 (1.13, 3.62) Delay: 2.56 (1.13, 5.80)
AIC	749.75	740.52	679.23	659.66

AIC: Akaike Information Criterion.
